# Stroke Damage Detection Using Classification Trees on Electrical Bioimpedance Cerebral Spectroscopy Measurements

**DOI:** 10.3390/s130810074

**Published:** 2013-08-07

**Authors:** Seyed Reza Atefi, Fernando Seoane, Thorleif Thorlin, Kaj Lindecrantz

**Affiliations:** 1 School of Technology and Health, Royal Institute of Technology, Alfred Nobels Allé 10, Huddinge SE-141 52, Sweden; E-Mails: fsm@kth.se (F.S.); kaj.lindecrantz@sth.kth.se (K.L.); 2 School of Engineering, University of Boras, Allégatan 1, Boras SE-501 90, Sweden; 3 Institute of Neuroscience and Physiology, Sahlgrenska Academy, University of Gothenburg, Gothenburg SE-405 30, Sweden; E-Mail: Thorleif.Thorlin@neuro.gu.se; 4 Department of Clinical Science, Intervention and Technology, Karolinska Institutet, Hälsovägen 7, Stockholm SE-141 57, Sweden

**Keywords:** stroke, electrical bioimpedance spectroscopy, classification tree, cole parameters

## Abstract

After cancer and cardio-vascular disease, stroke is the third greatest cause of death worldwide. Given the limitations of the current imaging technologies used for stroke diagnosis, the need for portable non-invasive and less expensive diagnostic tools is crucial. Previous studies have suggested that electrical bioimpedance (EBI) measurements from the head might contain useful clinical information related to changes produced in the cerebral tissue after the onset of stroke. In this study, we recorded 720 EBI Spectroscopy (EBIS) measurements from two different head regions of 18 hemispheres of nine subjects. Three of these subjects had suffered a unilateral haemorrhagic stroke. A number of features based on structural and intrinsic frequency-dependent properties of the cerebral tissue were extracted. These features were then fed into a classification tree. The results show that a full classification of damaged and undamaged cerebral tissue was achieved after three hierarchical classification steps. Lastly, the performance of the classification tree was assessed using Leave-One-Out Cross Validation (LOO-CV). Despite the fact that the results of this study are limited to a small database, and the observations obtained must be verified further with a larger cohort of patients, these findings confirm that EBI measurements contain useful information for assessing on the health of brain tissue after stroke and supports the hypothesis that classification features based on Cole parameters, spectral information and the geometry of EBIS measurements are useful to differentiate between healthy and stroke damaged brain tissue.

## 1. Introduction

Stroke is not only the third greatest cause of death worldwide [1] but also the cause of severe suffering to the surviving individuals with neurological deficits and a significant economic burden to society. The cost burden associated with post-stroke deficits has been estimated to be 65.5 billion USD for 2008 for the United States alone [2,3]. Stroke patients usually require the prompt intervention of clinical staff to prevent permanent lesions from developing. A time window of 3–4.5 h is usually required for certain treatments after the onset of stroke to maximise the treatment benefits and avoid permanent neurological impairment [4,5]. Every effort should be made to shorten the delay of the onset of therapy following stroke. Even a few minutes can make a difference. Adequate treatment within 90 min or less increases the probability of a favourable outcome for the patient [6]. However, many diagnostic tools, such as magnetic resonance imaging, X-ray or computer tomography are not accessible until patients reach a hospital, thus delaying the initiation of proper neural rescue therapies.

Moreover, none of these medical imaging devices can be used for continuous bedside monitoring of the brain. Therefore, to enable early detection of stroke and maximise the treatment outcome for the patients, it is crucial to have access to new monitoring tools that allow bedside monitoring or that can be used in ambulances.

Electrical bioimpedance (EBI) is a well-known, portable, affordable and non-invasive technology. Its use in various clinical activities and in physiological research has long been widespread [7–9]. Early applications of EBI used single-frequency measurements. Examples of these applications include impedance cardiography in 1940 [10] and lung function monitoring [11] in the 1970s. More recently, applications based on EBI Spectroscopy (EBIS) analysis have entered fields such as skin cancer detection [12] and nutritional status assessment in haemodialysis patients [13].

Since the 1950s and 1960s, EBI studies of the brain have been used to study different pathologies, including spreading depression, seizure activity, asphyxia and the effects of cardiac arrest [14]. Since this time, EBI research studies of the brain have been widespread, especially during the past 20 years [15,16]. Since Holder foresaw the development of electrical bioimpedance-based neurological applications in 1988 [16], several bioimpedance research and clinical studies have been performed in the areas of brain ischemia [17–19], spreading depression [20,21], epilepsy [21–24], brain function monitoring [25], perinatal asphyxia [26–28], cerebral blood flow monitoring [29–31] and stroke [32–34].

Stroke can be categorised into two main groups, namely, ischemia and haemorrhagic, with 87% of cases being ischemic and the remainder haemorrhagic [35]. Ischemic stroke is caused by arterial embolism or thrombosis, with a lack of blood supply to the brain followed by oedema, whereas haemorrhage is caused by accumulation of blood due to rupture of a blood vessel forming a haematoma. Different authors have drawn attention to the changes imposed on the electrical properties of the brain after an incident of cerebrovascular damage and have shown that these changes are detectable by means of electrical bioimpedance [16,32,36,37]. Both ischemia and haemorrhage will produce a change in the composition and structure of the cerebral tissue, and the electrical properties of the brain will change as a result [27,28,32,36–40].

It has been demonstrated that the electrical properties of the cerebral tissue change after an occurrence of stroke and that these changes are detectable from EBIS measurements performed on both hemispheres [37]. It has been observed that the Cole parameters [41] obtained from these EBIS measurements contain information that could be used to detect brain damage. However, this information was not sufficient to fully distinguish healthy cerebral tissue from stroke-damaged tissue.

Earlier studies have demonstrated the use of classification methods such as support vector machine (SVM) or multimodality classifier for detection of deteriorations in tissue or tissue characterization [42,43]. In this study, features extracted from EBIS measurements are fed into a relatively simple classification tree with the goal of separating healthy brain hemispheres from brain hemispheres with stroke damage. The authors believe the high explanatory power of classification tress make them an ideal choice for this study [44].

## 2. Materials and Methods

### 2.1. Tetrapolar EBI Measurements and Spectrometer

EBIS measurements were recorded using a SFB7 Spectrometer manufactured by Impedimed (Pinkenba, Queensland, Australia). Recordings were obtained in the frequency range of 3.096–1,000 kHz using the four electrode technique [45] and a sinusoidal current with the RMS value of 200 μA sweeping the frequency range over 256 logarithmically spaced frequency points. This will provide 256 complex EBI measurements from the frequency range. Two sets of EBIS measurements each containing 20 consecutive measurements were recorded from each hemisphere of all subjects. The first set includes measurements with the electrodes located close to the centre line of the head and the second set includes measurements with the electrodes located more laterally.

**Figure 1.**
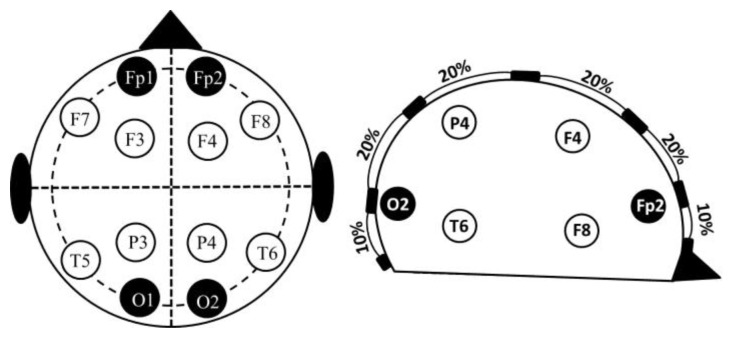
10–20 electrode placements system for performing the Cerebral EBIS measurements.

The electrodes were placed according to the 10–20 electrode placement system (Figure 1). The central measurements were labelled as M_CL_ or M_CR_ to indicate the left or right hemisphere. The lateral measurements were labelled as M_LL_ or M_LR_. All the recordings were performed using standard EEG silver electrodes dipped in Elefix electro-conductive paste. The details of electrode positioning are shown in Table 1. Prior to the application of the electrodes, the skin surface was prepared by cleaning the skin with alcohol and scrubbing gently with mild abrasive conductive cream.

### 2.2. Measurements Subjects

This study included a total of six volunteers and three patients who had suffered a unilateral stroke, *i.e.*, only one brain hemisphere was damaged by the stroke, The healthy volunteers were all males aged 29–54. The ages of the patients ranged from 40–55. Two of the patients were female, one male. The stroke was present in the left hemisphere in two patients and in the right hemisphere in the other patient. The patients were in the initial phase of rehabilitation, 6–8 weeks after the stroke episode. Details of the stroke location and relevant lesion area are listed in Table 2.

A total of 720 EBIS measurements were recorded from all nine subjects. From these measurements, 600 were recordings from 15 hemispheres with no stroke *i.e.*, undamaged and the remaining 120 were EBIS recordings from the three hemispheres with stroke. The 600 undamaged recordings consist of 480 EBIS recordings from twelve hemispheres of six healthy subjects and 120 measurements were from hemispheres of the patients with no stroke *i.e.*, undamaged hemispheres. This study was conducted with the ethical approval of the regional ethical review board of Gothenburg.

### 2.3. Cole Characterisation

Experimentally obtained EBI measurements can be fitted with sufficient accuracy by the Cole Equation (1), introduced by Cole in 1940 [41]:

(1) $${\text{Z}}_{\text{Cole}}(\text{f})={\text{R}}_{\infty }+\frac{{\text{R}}_{0}-{\text{R}}_{\infty }}{1+{\left(\overset{{\text{j}}^{\text{f}}}{\;}/\underset{{\text{f}}_{c}}{\;}\right)}^{\alpha }}\approx Z_{\text{Meas}}(\omega )$$

The Cole function consists of four parameters: *R_0_*, the *DC* resistance; *R_∞_*, the resistance at infinite frequency; *α*; and *f_c_*, the characteristic frequency [41,46]. In this study, all 720 EBIS measurements were fitted to the Cole model, and the Cole parameters were extracted for each measurement.

However, EBI measurements are known to be sensitive to stray capacitances in parallel with the measurement load, shifting the intrinsic dispersion of the tissue under study towards the dispersion of the capacitors in parallel and producing a deviation in the recorded data [47,48].

Seoane *et al.* have shown that the use of the real part of the admittance spectrum, *i.e.*, the conductance, to fit the Cole function using non-linear least squares (NLLS) fitting [49] in MATLAB^®^ allows the Cole parameters to be obtained free from capacitive leakage [50]. Therefore, as shown in [50], the value of the Cole parameters was estimated from the EBIS measurements based on NLLS fitting to the expression for the conductance Cole Equation (2) where Y_0_ and Y_∞_ are inverse of R_0_ and R_∞_, respectively:

(2) $$G_{\text{Cole}}(f)=Y_{0}+\frac{(Y_{\infty }-Y_{0})\left(1+\frac{Y_{\infty }}{Y_{0}}{\left(\overset{f}{\;}/\underset{f_{c}}{\;}\right)}^{-\alpha }\cos(\overset{\alpha \pi }{\;}/\underset{2}{\;})\right)}{1+\frac{Y_{\infty }^{2}}{Y_{0}^{2}}{\left(\overset{f}{\;}/\underset{f_{c}}{\;}\right)}^{-2\alpha }+2\frac{Y_{\infty }}{Y_{0}}{\left(\overset{f}{\;}/\underset{f_{c}}{\;}\right)}^{-\alpha }\cos(\overset{\alpha \pi }{\;}/\underset{2}{\;})}$$

### 2.4. Feature Set

Three features based on the Cole parameters, on spectral information and on the geometry of the EBIS measurements were defined for classification purposes. Each of these features was designed to be obtained from a different frequency region of the spectrum, *i.e.*, low, medium and high frequencies (see Table 3). To obtain the feature sets, the 720 measurements were first separated into two groups, namely, central and lateral, each consisting of 360 measurements.

The first two features were derived from Cole parameters R_0_ and R_∞_ as representatives of the high and low frequencies, respectively. These features were expressed as the ratio of central and lateral measurements inspired by the symmetry between brain hemispheres expected in a healthy subject. Earlier results reported suggest that these features will have a discriminative power around absolute value one [37]. The third feature was defined around the central frequency of the resistance and reactance spectra as the ratio of resistance to reactance at the characteristic frequency for the central and lateral measurements. This feature can be viewed as the phase of the impedance. In [51], it was proposed that this ratio is related to the ability of the volume fraction to track changes imposed on the tissue impedance. Therefore, this ratio is believed to contain useful information for monitoring a suspension of cells after changes are imposed on it due to the damaged mechanism, *i.e.*, ischemic or haemorrhagic damage.

Observing the Equation (3) for the electrical impedance of biological tissue as a shape factor usually related to length and area denoted by *k* and multiplied by its specific impedance z*(*ω*), known also as impedivity, Features 1 and 2 are primarily related to the shape factor, whereas Feature 3 targets the intrinsic frequency dependence:

(3) $$Z(\omega )=z^{*}(\omega )k=\int_{0}^{L}\frac{\mathrm{dx}}{S(x)}$$

### 2.5. Classification Trees

Reliable automatic decision making based on a set of simple questions is highly beneficial and greatly needed in medicine. In situations requiring a decision based on a large amount of input data, hierarchical decision-making algorithms can reduce the complexity of the problem and produce an accurate decision. Classification trees are hierarchical decision-making solutions that are well suited for the classification of categorical dependent variables. These solution methods offer a high degree of flexibility and exploratory power. Among other advantages of classification trees compared with other classification methods is their inherently non-parametric nature, which means they can be used regardless of the data distribution, whether normally distributed or skewed. In addition, classification trees have an inherent straightforward logic that is easily interpretable by a non-statistician or clinician. [52]. In a classification tree based on the attributes of a set of instances whose classes are already known, a mapping is performed from the attribute values to the classes, and the classification tree is essentially the formalisation of this mapping [44]. Each attribute/feature with the potential for classifying a data set will occupy a node of the tree, partitioning the data set into two subgroups, each placed on a leaf of the tree. This process continues until the tree is fully grown. Additionally, the convenient graphical display of classification trees is of substantial value in studying the performance of each individual attribute/feature more effectively.

In this study, three thresholds based on the features and symmetries were introduced hierarchically and placed at three nodes of a tree. Binary answers to the thresholds at each node were then used to separate the data into two new sub-classes, each placed on the leaves of the tree. The thresholds applied to Features 1 and 2 are essentially related to the geometry and shape factor of the EBI recordings derived from Cole parameters R_0_ and R_∞_, with an absolute value of approximately 1 according to the results stated in [37]. The threshold applied to Feature 3 was obtained experimentally.

### 2.6. Leave-One-Out Cross-Tree Validation

If the number of subjects is small, the measurements cannot be separated into two well-distributed groups of training and test data, as this split may produce an undesirable outcome and misleading results. In any case, other alternatives remain for evaluating the performance of the method in scenarios such as the current scenario.

At a higher computational cost, the Leave-One-Out (LOO) cross-validation technique [53] may be applied to provide useful values. In the application of the LOO method to an experiment *i.e.*, stroke detection with *N* examples *i.e.*, nine subjects in the case of this study, *N* iterations are performed with *N*–*1* examples used for training and 1 for testing. In each iteration *i*, the error *E_i_* and the final classification error are calculated using Equation (4). Note that *E_i_* is calculated as the percentage of misclassified measurements out of all measurements performed on one subject and a total of 40 measurements per subject (20 measurements per hemisphere) are provided, from each measurement three features are extracted:

(4) $$E=\frac{1}{N}\sum_{i=1}^{N}E_{i}$$

## 3. Results

### 3.1. Cole Parameters

**Figure 2.**
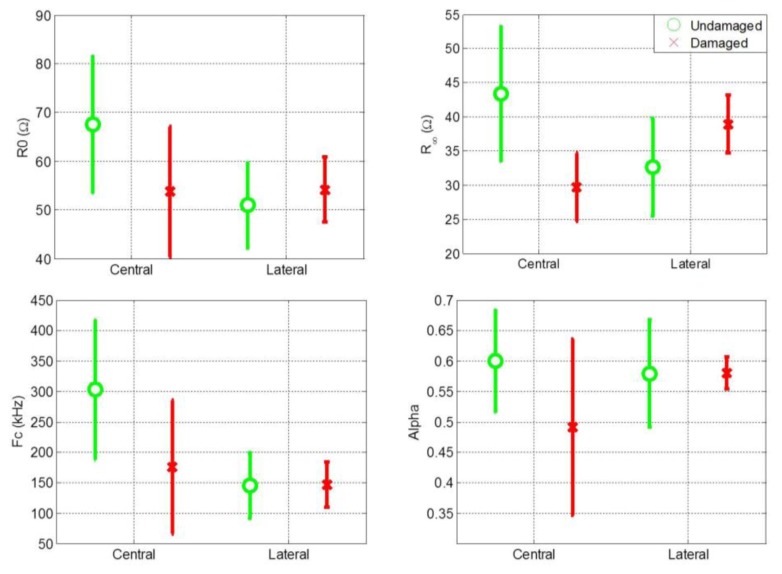
Mean and standard deviation of Cole parameters from all the EBIS measurements.

Cole parameters were extracted for all central and lateral measurements. The mean value and standard deviation of the Cole parameters for central and lateral measurements of both damaged and undamaged hemispheres are shown separately in Figure 2. Figure 2 shows that the distribution of the Cole parameter values exhibit a high variance. One reason for this high variance is the high variance among patients in the magnitude of the impedance recordings. It is also evident that comparing the Cole parameters individually for central and lateral measurements is not an effective procedure for distinguishing between healthy and damaged cerebral tissue.

### 3.2. Feature Maps

**Figure 3.**
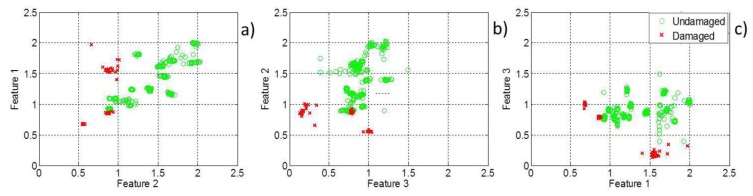
Feature maps for all the possible two-by-two combinations of the three features, plotted as two-dimensional maps.

The three features introduced in this study are plotted in a pairwise manner in two-dimensional feature maps, Figure 3. Figure 3 shows that feature Maps (a) and (b) can distinguish to a certain extent between healthy and damaged cerebral tissue but that a full classification is not possible based on either of these feature maps. However, a hierarchical decision-making classifier based on these features can fully separate stroke cases from healthy cases.

### 3.3. Classification Tree

**Figure 4.**
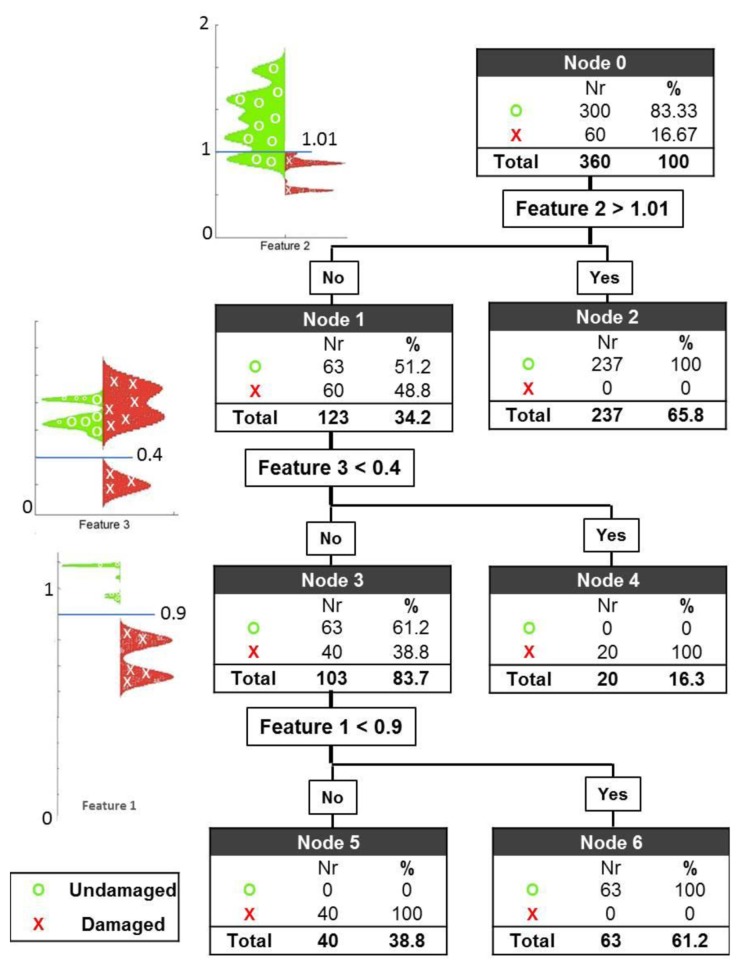
Classification tree. At each node, a threshold based on the features is applied, and the results are represented on the leaves of the tree.

The three features introduced earlier are situated at three different nodes of a classification tree with the following three thresholds: 0.9 for Feature 1, 1.10 for Feature 2 and 0.4 for Feature 3. The first and second thresholds are related to Cole parameters R_0_ and R_∞_ respectively as explained earlier they were expected to have values around 1 however the exact values were determined after the observation of feature maps in Figure 3. The third threshold was entirely selected by observation of feature maps. The results are shown on the leaves of the tree. The application of these thresholds yields a perfect classification of healthy and damaged cerebral tissue (see Figure 4).

At the first node, the first threshold is set to 1.01 for Feature 2 with the expectation of classifying a cluster of healthy subjects. With the application of this threshold, 79% of the healthy cases are correctly classified, leaving 21% of the healthy cases and 100% of the damage cases for further evaluation. Applying the second threshold with the value 0.4 for Feature 3, 1/3 of the damage cases are successfully separated, leaving 28.6% of all the measurements (63 healthy cases and 40 damage cases) for further evaluation. At the last node, the third and final threshold is then applied to Feature 1, with a value of 0.9. The result of this evaluation is that all 63 healthy cases are classified correctly in node 6, and all 40 remaining damaged cases are also correctly classified in node 5.

### 3.4. Classifier Performance

After a total of nine iterations implementing the LOO-CV test, not a single measurement was misclassified. In each iteration, all the data from the test set were separated perfectly into the healthy and damaged categories based on a classification tree customised with the values from the other eight data sets.

## 4. Discussion

Several previous studies have demonstrated the ability of electrical bioimpedance measurements at a single frequency of 50 kHz to detect haematoma and oedema as the principal indicators of stroke [33,34]. Although this approach is highly informative, EBIS measurements, in contrast to single-frequency EBI recordings, cover a wide range of frequencies from low to high, facilitating a more comprehensive study of all components within the tissue. Several previous studies have shown that EBIS measurements contain useful information on tissue characteristics and structure, thus providing features suitable for an automated classification of healthy/damaged tissue and, in particular, cerebral tissue [37,46,54]. It is known that impedance data are closely related to the geometry of the effective conductive volume and dielectric properties of the conductive tissue. For this reason, applying known geometrical information embedded in the EBIS recordings may produce more successful classification results [37].

In this study, we combined spectral information, *i.e.*, Cole parameters, with the measurement geometry to produce features that were well suited for classification. These features were then fed to a classification tree, and a perfect classification of healthy and damaged tissue was achieved after three steps. Due to the small size of the data set, which includes only three cases and six controls, the possibility that the results are due to chance cannot be excluded with a high degree of confidence. Moreover, the performance resulting from automatising the tree could not be evaluated. However, the results strengthen the hypothesis that cerebral EBIS can be useful in stroke detection and diagnosis. Given a sufficient number of subjects, the data set could be divided into two groups. The tree could be fully grown using the first data set. The performance of the classifier could then be tested with the second group. Lastly, pruning could be used to improve the accuracy of the classification tree [44]. Note, however, that the purpose of this manuscript is to claim that transencephalic EBIS measurements contain information on brain tissue state. This information is potentially useful for building a non-invasive monitoring device for the early detection of stroke damage.

## 5. Conclusions/Outlook

Three features were identified based on Cole parameters and geometry-related EBIS information. These were then fed into a simple classification tree that successfully separated healthy from stroke-damaged brain hemispheres. Although the available data were insufficient to perform a substantial evaluation of the performance of the classification approach or to apply other non-parametric automatic classification approaches, the results indicate that bioimpedance spectroscopy of head measurements provides useful information that might enable the development of a monitoring device to screen brain damage.

A larger data set including several patients should be obtained to verify the findings of this study and to allow the investigations to be extended to other classification modalities. Currently, transcephalic measurements of electrical bioimpedance are being performed on stroke patients at the Neurology ward at Karolinska Hospital. Accordingly, it is expected that these results will be verified in the near future with a larger cohort of patients.

## Figures and Tables

**Table 1.** Electrode positions according to 10–20 electrode positioning system.

	**M_LL_**	**M_CL_**	**M_CR_**	**M_LR_**
***I***^+^	Fp_1_	Fp_1_	Fp_2_	Fp_2_
***V***^+^	F_7_	F_3_	F_4_	F_8_
***V***^−^	T_5_	P_3_	P_4_	T_6_
***I***^−^	O_1_	O_1_	O_2_	O_2_

**Table 2.** Stroke location and size.

**Patient Number**	**Location**	**Size**
***i***	Putamen	6 × 3 cm
***ii***	Temporal Lobe	8.5 × 5.2 cm
***iii***	Tempoparietal Junction	4 × 4 cm

**Table 3.** Feature set.

**Feature 1**	**Feature 2**	**Feature 3**
$\frac{R0_{\text{Mcx}}}{R0_{\text{MLx}}}$	$\frac{R\infty_{\text{Mcx}}}{R\infty_{\text{MLx}}}$	$\frac{{\left(\overset{Rc}{\;}/\underset{Xc}{\;}\right)}_{\text{Mcx}}}{{\left(\overset{Rc}{\;}/\underset{Xc}{\;}\right)}_{\text{MLx}}}$
